# P-1514. Cefepime-taniborbactam Exhibits Limited Cross-resistance with Ceftazidime-avibactam and Ceftolozane-tazobactam against Carbapenem-nonsusceptible Enterobacterales and Multidrug-resistant *Pseudomonas aeruginosa* from the United States 2018-2022

**DOI:** 10.1093/ofid/ofae631.1683

**Published:** 2025-01-29

**Authors:** Meredith Hackel, Mark G Wise, Greg Moeck, Daniel F Sahm

**Affiliations:** IHMA, Schaumburg, Illinois; IHMA, Schaumburg, Illinois; Venatorx Pharmaceuticals, Malvern, Pennsylvania; IHMA, Schaumburg, Illinois

## Abstract

**Background:**

Cefepime-taniborbactam (FTB) is an investigational β-lactam/β-lactamase inhibitor combination with activity against most isolates of carbapenem-resistant and multidrug-resistant (MDR) Enterobacterales and *P. aeruginosa*. We evaluated the activity of FTB and comparators against clinical isolates of Enterobacterales and *P. aeruginosa* from the US and assessed FTB cross-resistance to ceftazidime-avibactam (CZA) and ceftolozane-tazobactam (CT) in resistant subsets.

**Figure:**
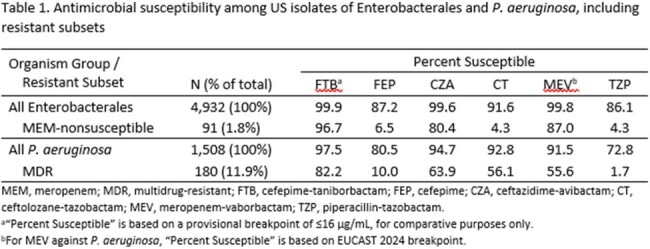

**Methods:**

MICs were determined by CLSI reference broth microdilution against Enterobacterales (n=4,932) and *P. aeruginosa* (n=1,508) collected in the US in 2018-2022. Phenotypes were based on 2024 CLSI breakpoints. A provisional FTB susceptible breakpoint of ≤16 µg/mL was used for comparative purposes. An MDR phenotype was defined as resistance to ≥1 agent from ≥3 drug classes.

**Figure:**
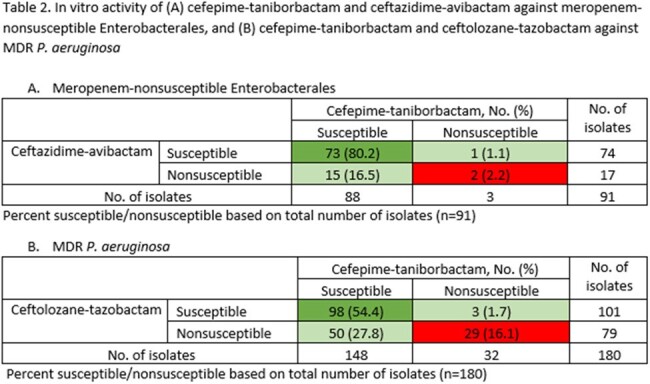

**Results:**

Among Enterobacterales, 1.8% of isolates were nonsusceptible to meropenem (MEM; Table 1). FTB was the most active agent, inhibiting 96.7% of MEM-nonsusceptible Enterobacterales isolates at ≤16 µg/mL whereas 80.4% were susceptible to CZA and 87.0% were susceptible to meropenem-vaborbactam (MEV). Among *P. aeruginosa*, 11.9% of isolates were MDR (Table 1). FTB was the most active agent, inhibiting 82.2% of MDR *P. aeruginosa* isolates at ≤16 µg/mL whereas 56.1% were susceptible to CT and 63.9% were susceptible to CZA (Table 1). Among MEM-nonsusceptible Enterobacterales, 80.2% were susceptible to both FTB and CZA, 16.5% were susceptible to FTB but not to CZA, 1.1% were susceptible to CZA but not to FTB, and 2.2% were nonsusceptible to both agents (Table 2A). Among MDR *P. aeruginosa*, 54.4% were susceptible to both FTB and CT, 27.8% were susceptible to FTB but not to CT, 1.7% were susceptible to CT but not to FTB, and 16.1% were nonsusceptible to both agents (Table 2B).

**Conclusion:**

FTB was active in vitro against recent clinical isolates of Enterobacterales and *P. aeruginosa* from the US including most isolates resistant to CZA and CT in key resistant subsets. These data support continued development of FTB as a potential treatment option for patients with challenging infections due to carbapenem-resistant Enterobacterales and MDR *P. aeruginosa*.

**Disclosures:**

**Greg Moeck, PhD**, Biomedical Advanced Research and Development Authority (BARDA): Grant/Research Support|Venatorx Pharmaceuticals, Inc.: Grant/Research Support|Venatorx Pharmaceuticals, Inc.: Stocks/Bonds (Private Company) **Daniel F. Sahm, PhD**, Pfizer, Inc.: Advisor/Consultant

